# Correlation and regression analysis of the *KRT27* and *ELOVL4* genes in cashmere fineness and other production performances in Liaoning cashmere goats

**DOI:** 10.5194/aab-68-135-2025

**Published:** 2025-02-12

**Authors:** Hua Ma, Weihang Hong, Lingjun Nie, Shuaitong Li, Qingyu Yuan, Ran Duan, Qiying Zhan, Lingchao Kong, Zeying Wang

**Affiliations:** 1 College of Animal Science & Veterinary Medicine, Shenyang Agricultural University, Shenyang, 110866, China; 2 Youth College of Political Science, Inner Mongolia Normal University, Hohhot, 010051, China

## Abstract

This research aims to explore how polymorphism in the *keratin 27* (*KRT27*) and *ELOVL fatty acid elongase 4* (*ELOVL4*) genes relates to the production traits of Liaoning cashmere goats (LCGs). PCR-seq was used to detect gene polymorphism in the experimental population DNA, and its correlations with production performance and regression analysis were calculated using the SPSS software. Single-nucleotide polymorphism (SNP) at locus 1919G/A was identified within *KRT27*, and the GG genotype showed superior wool fineness in doe, whereas the GA genotype showed favorable traits in buck. A SNP at locus 28666C/T was identified within *ELOVL4*, and the CC genotype showed excellent performance for cashmere fineness. The multi-genic effect genotype that affects the fineness of cashmere is a GGTT haplotype combination. Through multiple linear regression (MLR) analysis, it was found that the trait with the greatest direct impact on cashmere production performance and fineness is the cashmere yield rate, with a correlation coefficient of 0.915. The type with the greatest direct impact on lactation performance and cashmere fineness is TS, with a correlation coefficient of 16.369. The pleiotropism genotype that affects the fineness and related traits of cashmere has been determined to be the TT type. The GGTT haplotype combination, as an advantageous genotype that simultaneously affects cashmere fineness and lactation performance, can serve as a molecular marker of cashmere-fineness-assisted selection and provide a theoretical basis for the selection, breeding, and expansion of new fine-fiber strains of LCGs.

## 1 Introduction

Cashmere is a very precious fiber material produced by cashmere goats. Liaoning cashmere goats (LCGs) are the highest-producing white cashmere goat breed in the world (Diao et al., 2023; Wu et al., 2022b). They have the characteristics of long cashmere fiber, high net cashmere rate, moderate cashmere fineness, white cashmere, strong body size, strong adaptability, stable genetic performance, and good improvement effect on low- and medium-yield cashmere goats. LCGs are known as the national treasure of China. With LCGs as the original breed, five new local breeds have been cultivated, making outstanding contributions to the improvement and cultivation of Chinese cashmere goat breeds. In the cashmere industry, the fineness of cashmere is an important indicator of cashmere quality, which is related to the texture, comfort, and warmth of textiles (Jianhua et al., 2021). The Ministry of Agriculture and Rural Affairs of China has proposed the National Sheep Genetic Improvement Plan (2021–2035), which requires a 10 % increase in cashmere production and a reduction in cashmere fineness below 16 µ m, with room for further improvement. Therefore, reducing the diameter of its cashmere fibers is currently the direction for improving the quality of LCGs. Through spatial transcriptomic study of the fine and coarse skin tissues of cashmere in the early stage, we identified *KRT27* and *ELOVL4* genes with high differential expressions and potential regulatory effects on cashmere fineness. Therefore, we aim to investigate the effects of *KRT27* and *ELOVL4* on cashmere fineness through single-nucleotide polymorphism (SNP).

The *KRT* family is a gene family that encodes keratin intermediate filament (KIF), which is crucial in the keratinization process of animals, especially the development and structural characteristics of wool hair follicles. *KRT27* is a newly discovered member of the *KRT* family, and its expression in wool hair follicles exhibits a specific spatial pattern. This temporal expression pattern suggests that *KRT27* may play an important regulatory role in hair follicle cycle regulation, particularly in the growth and degeneration of hair follicles (Yu et al., 2009; Xiaolei et al., 2015). Zhang et al. (2024) found that *KRT27* is a factor that promotes hair growth, but its expression is inhibited by elevated homocysteine levels, thus forming part of the process of hair loss. Braun et al. (2018) discovered that the rs384881761 mutation in *KRT27* results in a curly hair phenotype in cattle. *KRT27* plays a pivotal role in determining hair morphology by secreting keratin, which ultimately shapes the final appearance of the hair (Shi et al., 2024). Nan et al. (2012) found that the expression of *KRT27* exhibited no significant difference in the heavily haired areas of sheep, whereas there was a highly significant variation in the expression of the sparsely haired areas. Juanjuan et al. (2012) discovered that *KRT27* expression is associated with the modulation of hair follicle density across various parts of fine-wooled sheep, potentially serving as a significant factor in regulating this density in these areas.

The *ELOVL* family plays a crucial role in maintaining cellular lipid metabolism balance, skin barrier function, and metabolic and inflammatory regulation (Wang et al., 2023). *ELOVL4* encodes an important enzyme class that primarily synthesizes very-long-chain fatty acids (VLCFAs). These VLCFAs play critical roles in various tissues, including the retina, brain, skin, and eyelid glands (Yeboah et al., 2021). *ELOVL4* gene mutations have been found to be associated with various diseases, such as Stargardt-like macular dystrophy, spinocerebellar ataxia, and neuroichthyosis (Hopiavuori et al., 2019). Wang et al. (2022) found that *ELOVL4* may affect muscle fat deposition in chickens by regulating the synthesis of long-chain unsaturated phospholipids. Agbaga et al. (2010) discovered that *ELOVL4* exhibits a prominent expression in the skin, concentrated particularly in the glandular epithelium of sebaceous glands and the surrounding tissue of hair follicle shafts. It plays a vital role in the epidermis, participating in the synthesis of very-long-chain fatty acids and producing omega-O-acylceramides, which are essential for forming an effective skin barrier (Vasireddy et al., 2008). Yongsheng et al. (2014) discovered that *ELOVL4* may be related to the fineness of cashmere.

The purpose of this research is to explore the impacts of SNP in the *KRT27* and *ELOVL4* genes of LCGs in terms of their cashmere production performance, body size performance, lambing performance, lactation performance, reproductive performance, slaughter performance, and meat quality performance. Through genetic diversity analysis and association analysis of six performance traits, the genotype and haplotype combinations that affect the six performance traits are determined, which is beneficial for breeding, and enhancement of LCG breeds advances the development of cashmere goat breeding endeavors and offers foundational justification for nurturing superior cashmere goat varieties.

## 2 Materials and methods

### 2.1 Experimental animals

This experiment selected the Liaoning Cashmere Goats Breeding Center in Liaoning Province for the collection of cashmere goat samples. A total of 1090 healthy and consistently fed cashmere goats were collected. All animal-handling methods and procedures used in this study were approved in accordance with the guidelines of the Experimental Animal Management Committee of Shenyang Agricultural University (Animal Welfare Ethics Certificate No. 2024.05.13). According to the guidance of a qualified veterinarian, 1 mL of a blood sample was collected from the jugular vein of each cashmere goat for DNA extraction. The collected blood was placed in a blood collection tube containing ethylene diamine tetraacetic acid (EDTA) and stored at -20 °C.

### 2.2 Production performance phenotype data

The performance data of cashmere goats are determined using a portable all-weather cashmere fineness and rapid-length testing machine. Cashmere samples are placed on the plate that comes with the analyzer, and once the plate is inserted into the analyzer the data can be obtained by clicking the “Start Test” button.

Body size data are collected from the intelligent body measurement system. A goat is directed into the instrument, and as it moves forward the system automatically detects its body measurements.

Lambing data are gathered using a counting method. During the lambing period, each doe's lambing events are counted and later summarized in a table.

Milk production data are acquired using a milk component analyzer. Two sample bottles are filled with milk from the same goat. One bottle is placed under the test port of the milk analyzer, while the pH sensor is immersed in the other bottle. After initiating the test, the results follow.

Slaughter data are obtained in compliance with the Operation Regulations for Slaughtering Poultry and Livestock (GB/T 43562-2023). All the data are collected through weighing and calculation processes.

The quality of the meat products is assessed based on the Technical Specification for Meat Quality Determination (T/CCAA 102-2023).

### 2.3 DNA extraction

Two-hundred milliliters of the blood sample was transferred to a centrifuge tube, followed by the addition of 20 µ L of Proteinase K mixed thoroughly until combined well. Next, 200 µ L of Buffer DL was added, shaken vigorously, and incubated in a water bath at 56 °C for 10 min. Subsequently, 200 µ L of anhydrous ethanol was introduced into the tube and mixed well. The mixture was poured into an adsorption column, allowed to stand for 2 min, and centrifuged at 10 000 rpm for 1 min at room temperature. The waste liquid was discarded in the collection tube, which was preceded by adding 500 µ L of GW solution to the adsorption column, centrifuging it at 10 000 rpm for 30 s, and discarding the waste liquid. This washing step was repeated with 700 µ L of wash solution, centrifuging at 10 000 rpm for 30 s, and discarding the waste liquid after each wash. This washing step was performed two additional times. Following the final wash, the adsorption column was centrifuged at 12 000 rpm for 2 min at room temperature to remove any residual liquid. The adsorption column was removed and placed in a fresh centrifuged tube. Capillary electrophoresis (CE) buffer (50 µ L) was added to the column, left to stand for 3 min, and centrifuged at 12 000 rpm for 2 min at room temperature. The eluted DNA solution and its optical density (OD) value were collected and measured using UV spectrophotometry. Qualifying samples were stored at -20 °C for future use.

### 2.4 Primer design

The *KRT27* and *ELOVL4* sequences were obtained from the NCBI database (NCBI reference sequences NC_030826.1 and NC_030816.1), and the customized primers were created with the assistance of the Premier 5 software application (Table 1).

**Table 1:** Primers were utilized for amplification and sequencing of *KRT27* and *ELOVL4* in LCGs.

Gene	Sense primer	TM (°)	Anti-sense primer	TM (°)	Fragment size	Region
*KRT27*	5^′^-CAGGAGACCAGCAGTGAA-3^′^	51.0	5^′^-GCCTTGTTATGAGCGATG-3^′^	51.7	947 bp	1521-2467(NC_030826.1)
*ELOVL4*	5^′^-ACATCAACATAAGTCAGCC-3^′^	47.0	5^′^-CACAACATTCCAATCGTAG-3^′^	48.7	981 bp	27892-28872(NC_030816.1)

### 2.5 PCR amplification

**Figure 1:**
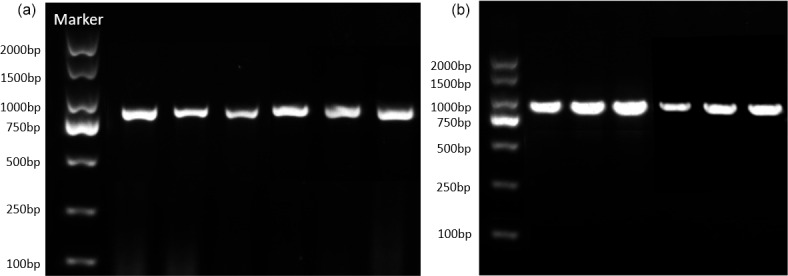
PCR amplification products of *KRT27* **(a)** and *ELOVL4* **(b)**.

The PCR reaction system has a total capacity of 50 µ L, comprising 25 µ L of 2× SanTaq PCR mix, 1 µ L of the DNA template, 2 µ L each of the upstream and downstream primers, and the remaining 20 µ L filled with ddH_2_O. These reagents are then added to the PCR tube, mixed thoroughly, centrifuged, and subsequently amplified in the PCR instrument based on the specified reaction conditions. The reaction proceeds with an initial denaturation step at 94 °C for 5 min, followed by denaturation at 94 °C for 30 s, annealing at 51 °C for 30 s, and extension at 72 °C for 1 min. This is concluded with a final extension step at 72 °C for 10 min. After electrophoresis, observe whether the bands in the electrophoresis results are qualified (Fig. 1). If they are qualified, send the sample to Shanghai Shenggong Biotechnology Co., Ltd. for sequencing.

### 2.6 Statistical analysis

Determine the genotype and allele frequencies and calculate the polymorphism information content (PIC), effective number of alleles ( $N_{e}$ ), and heterozygosity ( $H_{e}$ ). Single-factor analysis of the *KRT27* gene and *ELOL4* gene was conducted using the SPSS software in relation to the fineness and other traits of LCGs. Perform multiple comparisons using Duncan's method. The results are displayed as “mean ± SE”. The animal statistical model used is $Y_{ijkl}=\mu +h_{i}+p_{j}+s_{k}+m_{l}+e_{ijkdl}$ .

## 3 Results

### 3.1 SNP locus-sequencing map

**Figure 2:**
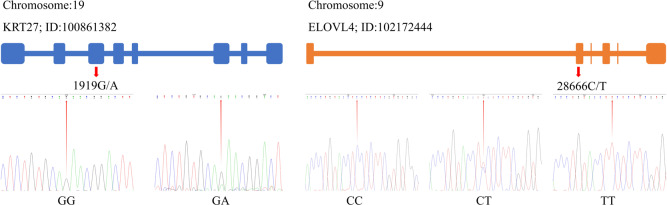
SNP identification in *KRT27* and *ELOVL4*.

By utilizing the Chromas 2 and DNAMAN software, we compared the obtained sequencing data with the gene sequences for *KRT27* and *ELOVL4*. This comparison revealed the presence of a SNP at locus 1919G/A within *KRT27*, and, additionally, a SNP at locus 28666C/T was identified in *ELOVL4* (Fig. 2).

### 3.2 Genetic diversity of *KRT27* and *ELOVL4*

Table 2 displays the genotype and allele frequencies of the SNP sites within *KRT27* and *ELOVL4* in LCGs. A gene is considered dominant if its frequency exceeds 0.5. For the 1919G/A locus of *KRT27* in LCGs, the PIC ranges between 0.25 and 0.5, indicating moderate polymorphism. The PIC of locus 28666C/T of *ELOVL4* in LCGs is between 0.25 and 0.5, indicating moderate polymorphism. The $\chi^{2}$ value of the 1919G/A and 28666C/T loci is greater than 1, and the p value is less than 0.05, which does not conform to the Hardy–Weinberg law and significantly deviates from the expected genetic frequency. This may be due to the fact that this group has already undergone manual selection, which may affect gene and genotype frequencies.

**Table 2:** Genetic character of the SNPs of the *KRT27* and *ELOVL4* genes.

Gender	Gene	Gene locus	Genotype frequency			Allele frequency		PIC	$H_{e}$	$N_{e}$	$X^{2}$	$P^{2}$
Doe	*KRT27*	1919G/A	GG(357)	GA(663)	AA(0)	G	A	0.34	0.44	1.78	236.46	$2.32493\times 10^{-53}$
			0.35	0.65	0	0.68	0.33					
	*ELOVL4*	28666C/T	CC(272)	CT(384)	TT(368)	C	T	0.37	0.50	1.98	60.64	$6.85147\times 10^{-15}$
			0.27	0.38	0.36	0.45	0.55					
Buck	*KRT27*	1919G/A	GG(20)	GA(32)	AA(0)	G	A	0.34	0.43	1.74	10.27	$1.35093\times 10^{-3}$
			0.38	0.62	0	0.69	0.31					
	*ELOVL4*	28666C/T	CC(18)	CT(18)	TT(30)	C	T	0.37	0.48	1.94	12.54	$3.98240\times 10^{-4}$
			0.27	0.27	0.45	0.41	0.59					

### 3.3 Gene substitution effect analysis of the *KRT27* and *ELOVL4* genes

From Table 3, it can be seen that the additive effect at the 1919G/A locus of the *KRT27* gene is negative, indicating that the mutation at this locus reduces the production performance of Liaoning cashmere goats and can decrease the fineness of cashmere, which is a positive effect. The additive effect of the *ELOVL4* gene 28666C/T locus is positive, but it is negative for cashmere fineness. Therefore, in terms of reducing cashmere fineness, the substitution effect of the *KRT27* gene 1919G/A locus is relatively good.

**Table 3:** Gene substitution effect analysis of the SNPs of the *KRT27* and *ELOVL4* genes.

Gender	Gene	Gene locus	Dominant	Additive	B gene	A gene	Average effect of
			effect	effect	average effect	average effect	B instead of A
Doe	*KRT27*	1919G/A	484.50	-178.50	-6.02	2.90	-8.92
	*ELOVL4*	28666C/T	64.00	48.00	19.03	-22.97	42.00
Buck	*KRT27*	1919G/A	22.00	-10.00	-1.07	0.47	-1.54
	ELLOVL4	28666C/T	-6.00	6.00	2.90	-4.19	7.09

### 3.4 Analysis of the cashmere performance of the *KRT27* and *ELOVL4* genes in LCGs

At the 1919G/A locus in LCG doe, the GA genotype exhibited superior length and length variation compared to the GG genotype, while the GG genotype showed finer cashmere than the GA genotype. Additionally, the GG genotype surpassed the GA genotype in curl count and short-fiber rate. At the 28666C/T locus, the CT genotype stood out, with a higher shear volume and better length variation than both CC and TT. The CC genotype, on the other hand, excelled in curl count and short-fiber rate over TT and CT (with CT better than TT for the short-fiber rate). The CC and TT types had a higher velvet yield compared to CT, while CC and TT were also finer in cashmere than CT.

In buck at the 1919G/A locus, the GG genotype demonstrated a significantly higher shear amount than GA, whereas GA was finer in cashmere quality. At the 28666C/T locus, CT had a notably higher shear volume than CC. For fiber length, CT outperformed both TT and CC, with TT being superior to CC. CC excelled in curl count over CT. In terms of the short-fiber rate, both CT and TT were better than CC. CC had a higher velvet yield compared to CT and TT. Notably, CC exhibited the finest cashmere of the three, significantly better than both CT and TT (Table 4).

**Table 4:** Analysis of the cashmere performance of the *KRT27* and *ELOVL4* genes.

Gender	Gene	Gene locus	Genotype	Cashmere quantity	Fineness	Natural length	Coefficient of variation	Number of	Short-fiber	Cashmere
				(g)	( µ m)	(mm)	of the length (%)	crimps	content (%)	rate (%)
Doe	*KRT27*	1919G/A	GG(208/1014)	1762.50 ± 14.62	16.30 ± 0.06^b^	92.88 ± 2.12^bB^	59.40 ± 0.79^aA^	5.35 ± 0.33^bB^	23.84 ± 1.14^aA^	77.67 ± 0.80
			GA(806/1014)	1788.71 ± 11.23	16.72 ± 0.05^a^	100.14 ± 1.06^aA^	52.63 ± 0.44^bB^	7.15 ± 0.13^aA^	15.08 ± 0.45^bB^	73.88 ± 5.42
	*ELOVL4*	28666C/T	CC(261/990)	1760.53 ± 15.89^bB^	16.46 ± 0.07^bB^	99.8 ± 1.58	50.55 ± 0.67^bB^	6.34 ± 0.23^b^	12.01 ± 0.69^cC^	76.09 ± 0.89^bA^
			CT(360/990)	1907.69 ± 14.58^aA^	17.03 ± 0.06^aA^	98.18 ± 1.7	49.04 ± 0.63^bB^	6.64 ± 0.20^ab^	15.42 ± 0.72^bB^	68.72 ± 0.66^cB^
			TT(369/990)	1645.45 ± 13.75^cC^	16.49 ± 0.07^bB^	96.28 ± 1.44	60.61 ± 0.57^aA^	7.05 ± 0.23^a^	18.74 ± 0.65^aA^	78.36 ± 0.82^aA^
Buck	*KRT27*	1919G/A	GG(20/50)	2035.00 ± 45.44^a^	16.57 ± 0.16	92.7 ± 2.74	56.41 ± 2.00	7.51 ± 0.62	16.42 ± 1.90	77.27 ± 1.70
			GA(30/50)	1900.00 ± 52.67^b^	16.39 ± 0.16	93.2 ± 2.86	55.93 ± 1.63	6.95 ± 0.47	20.90 ± 2.40	75.96 ± 2.00
	*ELOVL4*	28666C/T	CC(15/63)	1870.00 ± 72.78^b^	15.37 ± 0.22^cB^	79.12 ± 3.5^cC^	55.90 ± 2.48	6.22 ± 0.95^b^	37.64 ± 3.41^aA^	88.10 ± 2.20^aA^
			CT(18/63)	2066.67 ± 67.91^a^	16.61 ± 0.18^bA^	101.5 ± 1.5^aA^	61.33 ± 1.26	8.35 ± 0.15^a^	14.41 ± 0.73^bB^	74.50 ± 2.08^bB^
			TT(30/63)	1985.00 ± 39.71^ab^	17.14 ± 0.11^aA^	90.16 ± 2.14^bB^	56.22 ± 1.78	7.01 ± 0.32^ab^	14.94 ± 1.98^bB^	71.31 ± 1.80^bB^

Note: the expression method with no significant difference ( p>0.05 ) is the same shoulder letter, the expression method with a significant difference ( p<0.05 ) is the shoulder letter with different lowercase letters, and the expression method with an extremely significant difference ( p<0.01 ) is the shoulder letter with different uppercase letters.

### 3.5 Analysis of the body size performance of the *KRT27* and *ELOVL4* genes in LCGs

At the 1919G/A locus in LCG doe, the GG genotype significantly outperformed the GA genotype in body height, waist angle width, chest circumference, and waist height. For chest depth, GG was superior, while GA excelled in tube circumference. At the 28666C/T locus, TT was superior in body height to CT, while CC was superior in sacral height to TT. CC also significantly outperformed both CT and TT in chest depth and waist angle width. Notably, CC was superior in chest width to TT, while TT was superior in chest circumference to CC. In tube circumference, CT was superior to CC, and TT surpassed both CC and CT in waist height.

At the 1919G/A locus in LCG buck, the GG genotype significantly outperformed the GA genotype in sacral height and body length. However, GA excelled extremely significantly in chest depth compared to GG. GG was superior in chest width and waist angle width to GA. At the 28666C/T locus, both the CC and TT genotypes were very significantly superior to CT in body height and chest width. TT significantly surpassed CC in sacral height and body length. Both TT and CT were significantly superior to CC in chest depth. CC showed extreme significance in waist angle width and chest circumference compared to both TT and CT, with TT also being far superior to CT. In tube circumference, TT was far superior to both CC and CT, while CC was also far superior to CT (Table 5).

**Table 5:** Analysis of the body size performances of the *KRT27* and *ELOVL4* genes.

Gender	Gene	Gene locus	Genotype	Body height	Sacral height	Body oblique	Chest depth	Chest width	Hip width	Chest circumference	Shank circumference	Waist height
				(cm)	(cm)	(cm)	(cm)	(cm)	(cm)	(cm)	(cm)	(cm)
Doe	*KRT27*	1919G/A	GG(336/992)	64.62 ± 0.17^aA^	65.39 ± 0.21	78.30 ± 0.32	32.77 ± 0.16^a^	23.96 ± 0.21	23.77 ± 0.27^aA^	102.56 ± 0.57^aA^	9.35 ± 0.04^b^	65.45 ± 0.24^aA^
			GA(656/992)	63.32 ± 0.13^bB^	65.71 ± 0.12	78.61 ± 0.26	32.07 ± 0.10^b^	23.20 ± 0.16	22.06 ± 0.16^bB^	97.48 ± 0.30^bB^	9.59 ± 0.04^a^	62.95 ± 0.17^bB^
	*ELOVL4*	28666C/T	CC(261/990)	63.56 ± 0.18^ab^	65.73 ± 0.18^a^	79.09 ± 0.33	32.87 ± 0.17^aA^	24.60 ± 0.25^aA^	24.02 ± 0.31^aA^	98.67 ± 0.62^bB^	9.34 ± 0.05^bB^	63.61 ± 0.25^bB^
			CT(360/990)	63.39 ± 0.17^b^	65.27 ± 0.18^ab^	79.04 ± 0.28	32.10 ± 0.16^bB^	23.89 ± 0.20^abAB^	22.89 ± 0.22^bB^	99.65 ± 0.44^abAB^	9.57 ± 0.05^aA^	63.58 ± 0.23^bB^
			TT(369/990)	63.90 ± 0.16^a^	65.11 ± 0.15^b^	78.66 ± 0.34	32.32 ± 0.11^bB^	23.19 ± 0.20^cB^	22.33 ± 0.21^bB^	100.58 ± 0.44^aA^	9.49 ± 0.05^aAB^	64.60 ± 0.21^aA^
Buck	*KRT27*	1919G/A	GG(20/52)	75.00 ± 0.44	73.40 ± 0.50^a^	88.05 ± 0.66^a^	33.92 ± 0.67^bB^	28.28 ± 0.86^aA^	21.43 ± 0.44^aA^	104.50 ± 0.71	12.90 ± 0.21	68.60 ± 0.80
			GA(32/52)	74.93 ± 0.45	72.19 ± 0.38^b^	83.66 ± 1.98^b^	36.72 ± 0.28^aA^	24.88 ± 0.54^bB^	19.95 ± 0.41^bB^	105.43 ± 0.60	12.69 ± 0.11	69.13 ± 0.42
	*ELOVL4*	28666C/T	CC(18/66)	75.25 ± 0.78^aA^	71.33 ± 0.78^b^	78.92 ± 4.39^bB^	34.50 ± 0.94^b^	29.42 ± 1.02^aA^	23.33 ± 0.58^aA^	108.43 ± 0.41^aA^	12.75 ± 0.22^bB^	69.25 ± 0.67
			CT(18/66)	72.97 ± 0.34^bB^	72.17 ± 0.58^ab^	82.25 ± 0.95^bAB^	36.17 ± 0.20^a^	21.80 ± 0.24^bB^	18.17 ± 0.20^cC^	101.67 ± 0.79^cC^	11.92 ± 0.08^cC^	67.83 ± 0.58
			TT(30/66)	75.05 ± 0.43^aA^	73.15 ± 0.33^a^	90.15 ± 0.44^aA^	36.27 ± 0.42^a^	27.60 ± 0.69^aA^	20.90 ± 0.40^bB^	104.68 ± 0.71^bB^	13.45 ± 0.14^aA^	68.10 ± 0.64

Note: the expression method with no significant difference ( p>0.05 ) is the same shoulder letter, the expression method with a significant difference ( p<0.05 ) is the shoulder letter with different lowercase letters, and the expression method with an extremely significant difference ( p<0.01 ) is the shoulder letter with different uppercase letters.

### 3.6 Analysis of the lambing performance of the *KRT27* and *ELOVL4* genes in LCGs

Regarding the 1919G/A locus in LCGs, the GA genotype demonstrates notably higher lamb production compared to the GG genotype. At the 28666C/T locus, both the TT and CT genotypes exhibit significantly superior litter sizes in comparison to the CC genotype (Table 6).

**Table 6:** Analysis of the lambing performance of the *KRT27* and *ELOVL4* genes in LCGs.

Gene	Gene locus	Genotype	Lambing
*KRT27*	1919G/A	GG(123/363)	0.61 ± 0.04^bB^
		GA(240/363)	1.08 ± 0.03^aA^
*ELOVL4*	28666C/T	CC(90/306)	1.1 ± 0.03^bB^
		CT(117/306)	1.23 ± 0.03^aA^
		TT(99/306)	1.23 ± 0.04^aA^

Note: the expression method with no significant difference ( p>0.05 ) is the same shoulder letter, the expression method with a significant difference ( p<0.05 ) is the shoulder letter with different lowercase letters, and the expression method with an extremely significant difference ( p<0.01 ) is the shoulder letter with different uppercase letters.

### 3.7 Analysis of the milk performance of the *KRT27* and *ELOVL4* genes in LCGs

At the 1919G/A locus in LCGs, the fat content in the GA genotype was far superior to that of the GG genotype, while Cru.Prot and SnF were extremely significantly higher in GG than in GA. Cond. was significantly better in GG than in GA. At the 28666C/T locus, the fat content in the CT and TT genotypes was extremely significantly higher than in CC. Cru.Prot in CT was extremely significantly superior to both TT and CC, with TT also being far superior to CC. Lactose in CC was extremely significantly higher than in TT and CT, with TT being significantly superior to CT. Urea and N levels in CC were extremely significantly higher than in both CT and TT, with TT being significantly superior to CT. SnF in CT was extremely significantly superior to both TT and CC, with TT being significantly superior to CC. TS in CT was extremely significantly superior to both TT and CC, with TT being far superior to CC. Cond. in CT was extremely significantly superior to both TT and CC. H.Index in both CT and TT was extremely significantly higher than in CC (Table 7).

**Table 7:** Analysis of the milk performance of the *KRT27* and *ELOVL4* genes.

Gene	Gene locus	Genotype	Fat	Cru.Prot	Lactose	Urea	N	SnF	TS	Cond.	H.Index
*KRT27*	1919G/A	GG(169/299)	6.79 ± 0.11^bB^	5.91 ± 0.15^aA^	5.04 ± 0.04	40.87 ± 0.43	19.07 ± 0.20	11.62 ± 0.15^aA^	18.83 ± 0.16	778.56 ± 5.57^a^	0.45 ± 0.01
		GA(130/299)	7.90 ± 0.11^aA^	4.97 ± 0.07^bB^	5.14 ± 0.02	41.44 ± 0.25	19.33 ± 0.12	10.68 ± 0.05^bB^	18.96 ± 0.13	751.10 ± 2.55^b^	0.47 ± 0.00
*ELOVL4*	28666C/T	CC(63/280)	6.69 ± 0.12^cB^	4.49 ± 0.02^cC^	5.33 ± 0.01^aA^	40.56 ± 0.33^aA^	18.92 ± 0.15^aA^	10.31 ± 0.02^cB^	17.32 ± 0.13^cC^	750.56 ± 3.34^bB^	0.43 ± 0.00^Bb^
		CT(91/280)	7.56 ± 0.13^aA^	5.68 ± 0.13^aA^	4.90 ± 0.06^cB^	38.99 ± 0.39^bB^	18.19 ± 0.18^bB^	11.23 ± 0.11^aA^	19.23 ± 0.17^aA^	796.42 ± 9.79^aA^	0.50 ± 0.01^aA^
		TT(126/280)	7.14 ± 0.11^bA^	4.84 ± 0.07^bB^	5.20 ± 0.02^Ba^	39.92 ± 0.22^aAB^	18.63 ± 0.11^aAB^	10.57 ± 0.06^bB^	18.06 ± 0.14^bB^	765.99 ± 3.04^bB^	0.48 ± 0.00^aA^

Note: the expression method with no significant difference ( p>0.05 ) is the same shoulder letter, the expression method with a significant difference ( p<0.05 ) is the shoulder letter with different lowercase letters, and the expression method with an extremely significant difference ( p<0.01 ) is the shoulder letter with different uppercase letters.

### 3.8 Analysis of the slaughter performance of the *KRT27* and *ELOVL4* genes in LCGs

At the 1919G/A locus in LCG doe, the GA genotype significantly outperformed the GG genotype in carcass weight, while the GG genotype was far superior to the GA genotype in net meat weight, dressing percentage, net meat percentage, carcass net meat percentage, GR value, and back-fat thickness. The GA genotype was far superior to the GG genotype in the eye muscle area. At the 28666C/T locus, the CC genotype was far superior to both the CT and TT genotypes in pre-slaughter live weight and carcass weight, with CT also being far superior to TT. For dressing percentage, the CT and TT genotypes were far superior to the CC genotype. For net meat rate and carcass net meat rate, TT was far superior to both CT and CC, with CT also being far superior to CC. The CT genotype was far superior to both TT and CC in eye muscle area and GR value. Both the CT and TT genotypes were far superior to CC in back-fat thickness.

At the 1919G/A locus in LCG buck, the GG genotype showed extremely significant superiority to the GA genotype in live weight before slaughter, carcass weight, net meat weight, net meat percentage, carcass net meat percentage, GR value, and back-fat thickness. However, the GA genotype was extremely significantly superior to the GG genotype in eye muscle area. At the 28666C/T locus, the CC genotype exhibited extremely significant superiority to the TT genotype in carcass weight and net meat weight. The CC genotype also significantly outperformed both the CT and TT genotypes in dressing percentage and net meat percentage, while the CT genotype was significantly better than the TT genotype. For carcass net meat percentage and back-fat thickness, the CC genotype was extremely significantly superior to both the CT and TT genotypes. In contrast, eye muscle area was significantly better in the CT and TT genotypes compared to the CC genotype (Table 8).

**Table 8:** Analysis of the slaughter performance of the *KRT27* and *ELOVL4* genes.

Gender	Gene	Gene locus	Genotype	Live weight before	Carcass weight	Net meat	Slaughter	Net meat	Carcass net	Eye muscle area	GR	Back-fat thickness
				slaughter (kg)	(kg)	weight (kg)	rate (%)	rate (%)	meat rate (%)	(cm^2^)	(mm)	(mm)
Doe	*KRT27*	1919G/A	GG(35/60)	45.90 ± 0.24	23.51 ± 0.15^a^	19.47 ± 0.13^aA^	51.17 ± 0.10^aA^	42.32 ± 0.11^aA^	82.65 ± 0.06^aA^	18.78 ± 0.05^bB^	8.23 ± 0.09^aA^	3.08 ± 0.04^aA^
			GA(25/60)	46.46 ± 0.32	22.60 ± 0.12^b^	18.36 ± 0.11^bB^	48.86 ± 0.08^bB^	39.66 ± 0.10^bB^	81.15 ± 0.11^bB^	20.48 ± 0.17^aA^	7.74 ± 0.05^bB^	2.50 ± 0.03^bB^
	*ELOVL4*	28666C/T	CC(10/60)	50.35 ± 0.55^aA^	23.65 ± 0.20^aA^	19.15 ± 0.16^aA^	47.21 ± 0.13^bB^	38.23 ± 0.10^cC^	80.98 ± 0.00^cC^	19.74 ± 0.09^bA^	7.52 ± 0.09^bB^	1.61 ± 0.00^cB^
			CT(35/60)	46.14 ± 0.24^bB^	23.47 ± 0.15^aB^	19.33 ± 0.15^aA^	50.71 ± 0.08^aA^	41.64 ± 0.10^bB^	82.07 ± 0.10^bB^	20.17 ± 0.12^aA^	8.47 ± 0.04^aA^	3.13 ± 0.03^aA^
			TT(15/60)	43.30 ± 0.28^cC^	22.00 ± 0.08^bB^	18.17 ± 0.06^bB^	51.04 ± 0.16^aA^	42.18 ± 0.14^aA^	82.63 ± 0.08^aA^	17.72 ± 0.03^cB^	7.34 ± 0.19^bB^	2.98 ± 0.04^bA^
Buck	*KRT27*	1919G/A	GG(14/28)	49.43 ± 0.71^aA^	25.64 ± 0.45^aA^	20.47 ± 0.43^aA^	51.81 ± 0.36	41.27 ± 0.39^aA^	79.61 ± 0.36^aA^	19.91 ± 0.16^bB^	7.44 ± 0.22^aA^	2.36 ± 0.15^aA^
			GA(14/28)	44.13 ± 0.30^bB^	22.46 ± 0.27^bB^	17.13 ± 0.24^bB^	50.88 ± 0.49	38.80 ± 0.45^bB^	76.20 ± 0.25^bB^	24.51 ± 0.66^aA^	5.18 ± 0.21^bB^	1.75 ± 0.06^bB^
	*ELLOVL4*	28666C/T	CC(6/24)	47.90 ± 0.46	26.17 ± 0.30^aA^	20.93 ± 0.41^aA^	54.60 ± 0.09^aA^	43.60 ± 0.43^aA^	79.81 ± 0.64^aA^	24.70 ± 0.79^bB^	6.69 ± 0.32	2.73 ± 0.25^aA^
			CT(4/24)	47.20 ± 1.41	24.70 ± 0.56^abAB^	19.50 ± 0.46^abAB^	52.53 ± 0.38^bB^	41.45 ± 0.26^bB^	78.92 ± 0.08^bB^	26.31 ± 1.62^aA^	6.43 ± 0.54	1.49 ± 0.05^bB^
			TT(14/24)	46.27 ± 0.73	23.46 ± 0.48^bB^	18.03 ± 0.44^bB^	50.57 ± 0.40^cC^	38.74 ± 0.41^cC^	76.53 ± 0.35^bB^	20.44 ± 0.28^aA^	6.18 ± 0.26	1.81 ± 0.10^bB^

Note: the expression method with no significant difference ( p>0.05 ) is the same shoulder letter, the expression method with a significant difference ( p<0.05 ) is the shoulder letter with different lowercase letters, and the expression method with an extremely significant difference ( p<0.01 ) is the shoulder letter with different uppercase letters.

### 3.9 Analysis of the meat performance of the *KRT27* and *ELOVL4* genes in LCGs

In LCG doe, at the 1919G/A locus, the L values of meat color and fat content were significantly higher in the GG genotype than in the GA genotype, while the b values of meat color, pH, dry matter content, drip loss, and cooked meat yield were significantly higher in the GA genotype. At the 28666C/T locus, the TT genotype exhibited significantly higher L values than the CT and CC genotypes, with CT being significantly higher than CC. The CT genotype had the highest value, followed by TT and CC. The CC genotype showed the highest b value, protein content, and fat content, with TT higher than CT. The CT genotype had the highest pH and drip loss, followed by TT and CC. The CC genotype had the highest cooked meat yield, followed by CT and TT. Shear force was highest in the CC and TT genotypes compared to CT.

In LCG buck, at the 1919G/A locus, the GG genotype had significantly higher a values for meat color and shear force than GA, while the pH and fat content were significantly higher in GG. The GA genotype showed significantly higher b values for meat color, protein content, drip loss, and cooked meat yield than GG. At the 28666C/T locus, the CT genotype had the highest L value compared to TT. The TT genotype exhibited a significantly higher a value than CT and CC. The CC genotype had the highest b value and pH, followed by CT and TT. The CT genotype had the highest protein content compared to TT. The TT genotype had the highest fat content, followed by CT and CC. The CC genotype showed the highest drip loss, followed by CT (significantly higher than TT). The TT genotype had the highest cooked meat yield, followed by CT and CC. The CC genotype had the highest shear force, followed by TT (both significantly higher than CT) (Table 9).

**Table 9:** Analysis of the meat performance of the *KRT27* and *ELOVL4* genes.

Gender	Gene	Gene locus	Genotype	Meat color			pH	Dry matter	Protein content	Fat content	Less drip	Cooked meat	Shear force
								(%)	(%)	(%)	(%)	rate (%)	(N)
				L	a	b							
Doe	*KRT27*	1919G/A	GG(35/60)	30.58 ± 0.09^aA^	14.20 ± 0.05	1.73 ± 0.02^bB^	5.93 ± 0.00^bB^	27.11 ± 0.08^bB^	20.79 ± 0.04	2.61 ± 0.05^aA^	1.62 ± 0.01^bB^	66.67 ± 0.20^bB^	70.37 ± 0.41
			GA(25/60)	28.90 ± 0.09^bB^	14.42 ± 0.08	1.98 ± 0.03^aA^	6.01 ± 0.01^aA^	28.94 ± 0.22^aA^	20.97 ± 0.06	1.91 ± 0.03^bB^	1.90 ± 0.01^aA^	71.03 ± 0.07^aA^	71.85 ± 0.41
	*ELOVL4*	28666C/T	CC(10/60)	27.55 ± 0.10^cC^	14.00 ± 0.10^bB^	2.30 ± 0.02^aA^	5.75 ± 0.00^cC^	26.40 ± 0.01^bB^	22.20 ± 0.04^aA^	3.30 ± 0.04^aA^	1.55 ± 0.03^cC^	70.80 ± 0.19^aA^	75.75 ± 0.84^aA^
			CT(35/60)	29.87 ± 0.09^bB^	14.63 ± 0.06^aA^	1.64 ± 0.02^cC^	6.04 ± 0.01^aA^	28.90 ± 0.17^aA^	20.47 ± 0.05^cC^	1.83 ± 0.03^cC^	1.81 ± 0.01^aA^	69.41 ± 0.13^bB^	67.34 ± 0.35^bB^
			TT(15/60)	31.50 ± 0.03^aA^	13.70 ± 0.05^cB^	2.00 ± 0.04^bB^	5.93 ± 0.00^bB^	26.47 ± 0.05^bB^	20.90 ± 0.05^bB^	2.73 ± 0.08^bB^	1.70 ± 0.01^bB^	64.77 ± 0.32^cC^	76.33 ± 0.28^aA^
Buck	*KRT27*	1919G/A	GG(14/28)	29.54 ± 0.24	15.30 ± 0.27^a^	1.64 ± 0.02^bB^	6.11 ± 0.02^aA^	25.82 ± 0.21	20.88 ± 0.13^bB^	2.13 ± 0.09^aA^	1.57 ± 0.03^bB^	64.83 ± 0.56^bB^	76.49 ± 0.86^a^
			GA(14/28)	29.23 ± 0.19	14.38 ± 0.17^b^	1.77 ± 0.06^aA^	6.02 ± 0.02^bB^	25.83 ± 0.17	22.00 ± 0.07^aA^	1.60 ± 0.08^bB^	1.75 ± 0.02^aA^	67.32 ± 0.32^aA^	73.42 ± 0.97^b^
	*ELLOVL4*	28666C/T	CC(6/24)	29.27 ± 0.31^bAB^	14.00 ± 0.11^bB^	2.10 ± 0.10^aA^	6.23 ± 0.03^aA^	25.83 ± 0.27	21.53 ± 0.35^ab^	1.17 ± 0.07^cC^	1.87 ± 0.01^aA^	63.70 ± 1.16^bB^	80.50 ± 1.13^aA^
			CT(4/24)	30.10 ± 0.29^aA^	13.45 ± 0.04^bB^	1.65 ± 0.01^bB^	6.10 ± 0.02^bB^	26.50 ± 0.36	21.90 ± 0.05^a^	1.65 ± 0.08^bB^	1.70 ± 0.02^bB^	64.40 ± 0.17^bB^	67.25 ± 0.21^cC^
			TT(14/24)	28.79 ± 0.20^bB^	15.43 ± 0.26^aA^	1.60 ± 0.02^bB^	6.01 ± 0.02^cB^	25.86 ± 0.19	21.27 ± 0.08^b^	2.17 ± 0.08^aA^	1.59 ± 0.03^cB^	67.49 ± 0.29^aA^	74.94 ± 0.88^bB^

Note: the expression method with no significant difference ( p>0.05 ) is the same shoulder letter, the expression method with a significant difference ( p<0.05 ) is the shoulder letter with different lowercase letters, and the expression method with an extremely significant difference ( p<0.01 ) is the shoulder letter with different uppercase letters.

### 3.10 Correlation analysis of cashmere fineness and cashmere performance in LCGs

According to Table 10, the shearing amount, cashmere length, length variation coefficient, curl number, short cashmere rate, and cashmere yield of LCGs and doe are all significantly correlated with the cashmere fineness. For buck, only the shearing amount, cashmere yield, and cashmere fineness are significantly correlated, while a short cashmere rate is significantly correlated with fineness. The order of correlation coefficients between wool fineness and wool production traits in doe is as follows: shearing amount > length > curl number > short wool rate > length variation coefficient > wool yield rate. For buck, the order is shearing amount > short wool rate > wool yield rate.

**Table 10:** Correlation analysis of cashmere fineness and cashmere performance in LCGs.

Buck	Doe						
	Fineness	Cashmere	Natural	Coefficient of	Number	Short-fiber	Cashmere
		quantity	length	variation of length	of crimps	content	rate
Fineness	–	0.347^∗∗^	0.343^∗∗^	$-0.312^{**}$	$-0.126^{**}$	$-0.149^{**}$	$-0.927^{**}$
Cashmere quantity	0.443^∗∗^	–	0.098^∗∗^	$-0.325^{**}$	$-0.116^{**}$	$-0.087^{**}$	$-0.333^{**}$
Natural length	0	$-0.275^{**}$	–	$-0.458^{**}$	0.049	$-0.365^{**}$	$-0.342^{**}$
Coefficient of variation of length	0.016	0.260^∗∗^	0.051	–	0.063	0.613^∗∗^	0.376^∗∗^
Number of crimps	-0.061	0.159	0.276^∗∗^	0.766^∗∗^	–	$-0.177^{**}$	0.125^∗∗^
Short-fiber content	$-0.229^{*}$	0.043	$-0.338^{**}$	0.157	$-0.301^{**}$	–	0.173^∗∗^
Cashmere rate	$-0.895^{**}$	$-0.288^{**}$	0.016	0.126	0.065	0.328^∗∗^	–

Note: the upper triangle represents the correlation analysis results for the fineness of doe cashmere and cashmere performance, while the lower triangle represents the correlation analysis results for the fineness of buck cashmere and cashmere performance. The shoulder markers ^**^ indicate extremely significant correlation ( P<0.01 ), ^*^ indicates significant correlation ( P<0.05 ), and no ^*^ indicates insignificant correlation ( P>0.05 ).

### 3.11 Path analysis of fineness and cashmere performance in LCGs

According to Table 11, the cashmere yield of LCGs has the greatest direct impact on the fineness of cashmere, which is 0.915. The second greatest is the coefficient of variation of length, and the third greatest is the amount of shearing. The indirect effect of length on fineness is greatest, at -0.061 , followed by the amount of shearing. The wool yield of buck has the greatest direct impact on the fineness of cashmere, which is 0.825, followed by the shearing amount and the short wool rate. The indirect impact of shearing amount on fineness is greatest, at -0.051 , followed by a short pile rate. From this, it can be seen that the cashmere yield and shearing amount are key traits that affect the fineness of cashmere.

**Table 11:** Path analysis of fineness and cashmere performance in LCGs.

Gender	Argument	Correlation	Direct diameter	Indirect path coefficient					
		coefficient	coefficient						
				Cashmere	Natural	Coefficient of	Number	Short-fiber	Cashmere
				quantity	length	variation of length	of crimps	content	rate
Doe	Cashmere quantity	0.347	0.064	–	0.006	-0.021	-0.007	-0.006	-0.021
	Natural length	0.343	0.06	0.006	–	-0.027	0.003	-0.022	-0.021
	Coefficient of variation of length	-0.312	0.098	-0.032	-0.045	–	0.006	0.060	0.037
	Number of crimps	-0.126	0.018	-0.002	0.001	0.001	–	-0.003	0.002
	Short-fiber content	-0.149	0.027	-0.002	-0.010	0.017	-0.005	–	0.005
	Cashmere rate	-0.927	0.915	-0.305	-0.313	0.344	0.114	0.158	–
Buck	Cashmere quantity	0.443	0.209	–	–	–	–	0.009	-0.060
	Short-fiber content	-0.229	0.104	0.004	–	–	–	–	0.034
	Cashmere rate	-0.895	0.825	-0.238	–	–	–	0.271	–

### 3.12 Stepwise multiple regression analysis results of cashmere performance and fineness in LCGs

The optimal regression equation was established based on the fineness of cashmere, together with the cashmere yield, shearing amount, coefficient of variation of length, and length. For doe, CF = -0.077CR+0.0003CY+0.008CV+0.002CL+21.268 , and for buck CF = -0.07CR+0.001CY+22.203 . The determination coefficients ( $R^{2}$ values) of the multiple regression equations were 0.865 and 0.839, respectively, indicating that this formula can explain the changes in cashmere fineness of 86.5 % and 83.9 % (Table 12).

**Table 12:** Results of stepwise multiple regression analysis of cashmere performance and fineness in LCGs.

Gender	Model	$R^{2}$	Adjusted $R^{2}$	Standard error	F	P
				of the estimate		
Doe	CF = -0.078CR+22.483	0.858	0.858	0.470	6137.864	<0.001
	CF = -0.077CR+0.0002CY+22.072	0.860	0.860	0.467	3108.241	<0.001
	CF = -0.079CR+0.0002CY+0.006CV+21.784	0.863	0.862	0.463	2114.995	<0.001
	CF = -0.077CR+0.0003CY+0.008CV+0.002CL+21.268	0.865	0.865	0.459	1622.383	<0.001
Buck	CF = -0.75CR+22.203	0.801	0.799	0.515	387.256	<0.001
	CF = -0.07CR+0.001CY+22.203	0.839	0.835	0.466	247.137	<0.001

Note: the dependent variable is the fineness of cashmere, CF is the fineness of cashmere, CY is the cashmere rate, and CR is the cashmere quantity. $R^{2}$ is the coefficient of determination, and the F value is the result of the F test.

### 3.13 Correlation analysis of cashmere fineness and milk performance in LCGs

According to Table 13, there is a highly significant correlation between urea, TS, and cashmere fineness in LCG doe, while fat, Cru.Prot, lactose, N , and SnF are significantly correlated with cashmere fineness. The order of the correlation coefficients for wool fineness and milk production traits in doe is TS > urea > N > fat > Cru.Prot > lactose > SnF.

**Table 13:** Correlation analysis of cashmere fineness and milk performance in LCGs.

	Fineness	Fat	Cru.Prot	Lactose	Urea	N	SnF	TS	Cond.	H.Index
Fineness	–	0.074^∗^	0.067^∗^	$-0.064^{*}$	$-0.080^{**}$	$-0.079^{*}$	0.062^∗^	0.088^∗∗^	0.022	-0.026
Fat	0.074^∗^	–	0.095^∗∗^	$-0.186^{**}$	0.575^∗∗^	0.574^∗∗^	0.055	0.852^∗∗^	$-0.319^{**}$	0.470^∗∗^
Cru.Prot	0.067^∗^	0.095^∗∗^	–	$-0.815^{**}$	0.263^∗∗^	0.263^∗∗^	0.955^∗∗^	0.585^∗∗^	0.556^∗∗^	0.092^∗∗^
Lactose	$-0.064^{*}$	$-0.186^{**}$	$-0.815^{**}$	–	$-0.332^{**}$	$-0.332^{**}$	$-0.609^{**}$	$-0.487^{**}$	$-0.810^{**}$	$-0.552^{**}$
Urea	$-0.080^{**}$	0.575^∗∗^	0.263^∗∗^	$-0.332^{**}$	–	10.000^∗∗^	0.185^∗∗^	0.575^∗∗^	0.02	0.239^∗∗^
N	$-0.079^{*}$	0.574^∗∗^	0.263^∗∗^	$-0.332^{**}$	10.000^∗∗^	–	0.185^∗∗^	0.574^∗∗^	0.021	0.239^∗∗^
SnF	0.062^∗^	0.055	0.955^∗∗^	$-0.609^{**}$	0.185^∗∗^	0.185^∗∗^	–	0.569^∗∗^	0.341^∗∗^	$-0.148^{**}$
TS	0.088^∗∗^	0.852^∗∗^	0.585^∗∗^	$-0.487^{**}$	0.575^∗∗^	0.574^∗∗^	0.569^∗∗^	–	$-0.066^{*}$	0.326^∗∗^
Cond.	0.022	$-0.319^{**}$	0.556^∗∗^	$-0.810^{**}$	0.02	0.021	0.341^∗∗^	$-0.066^{*}$	–	0.427^∗∗^
H.Index	-0.026	0.470^∗∗^	0.092^∗∗^	$-0.552^{**}$	0.239^∗∗^	0.239^∗∗^	$-0.148^{**}$	0.326^∗∗^	0.427^∗∗^	–

Note: the upper triangle represents the correlation analysis results for the fineness of doe cashmere and cashmere performance, while the lower triangle represents the correlation analysis results for the fineness of buck cashmere and cashmere performance. The shoulder markers ^**^ indicate extremely significant correlation ( P<0.01 ), ^*^ indicates significant correlation ( P<0.05 ), and no ^*^ indicates insignificant correlation ( P>0.05 ).

### 3.14 Path analysis of the fineness and milk performance in LCGs

Due to its high collinearity with other variables, N is excluded.

According to Table 14, the TS of LCG doe has the greatest direct impact on cashmere fineness, reaching 16.369, followed by fat and SnF. TS has the greatest indirect impact on fineness, with a value of 34.276, followed by fat and SnF. It can be seen that TS, fat, and SnF are key traits that affect the fineness of cashmere.

**Table 14:** Path analysis of fineness and milk performance in LCGs.

Argument	Correlation coefficient	Direct diameter coefficient	Indirect path coefficient					
			Fat	Cru.Prot	Lactose	Urea	SnF	TS
Fat	0.074	14.132	–	1.343	-2.629	8.126	0.777	12.040
Cru.Prot	0.067	3.635	0.345	–	-2.963	0.956	3.471	2.126
Lactose	-0.064	1.132	-0.211	-0.923	–	-0.376	-0.689	-0.551
Urea	-0.08	0.33	0.190	0.087	-0.110	–	0.061	0.190
SnF	0.062	5.625	0.309	5.372	-3.426	1.041	–	3.201
TS	0.088	16.369	13.946	9.576	-7.972	9.412	9.314	–

### 3.15 Stepwise multiple regression analysis results of milk performance and fineness in LCGs

The optimal regression equation based on cashmere fineness and TS/U is CF = 0.173TS-0.086U+15.972 . The coefficient of determination ( $R^{2}$ ) of the multiple regression equation is 0.033, indicating that this formula can explain the 3.3 % variation in cashmere fineness (Table 15).

**Table 15:** Stepwise multiple regression analysis results of milk performance and fineness in LCGs.

Model	$R^{2}$	Adjusted $R^{2}$	Standard error of the estimate	F	P
CF=0.076TS+14.337	0.008	0.007	2.481	8.041	0.005
CF=0.173TS-0.086U+15.972	0.033	0.031	2.450	17.767	<0.001

Note: the dependent variable is the cashmere fineness, CF is the cashmere fineness, $R^{2}$ is the coefficient of determination, and the F value is the result of the F test.

### 3.16 Haplotype combination analysis of the *KRT27* and *ELOVL4* genes

Using the SHEsis software (http://analysis.bio-x.cn/myAnalysis.php, last access: 10 July 2024), analysis of the SNP loci in the *KRT27* and *ELOVL4* genes revealed the formation of six haplotype combinations (Table 16).

**Table 16:** Haplotype combination analysis of the *KRT27* and *ELOVL4* genes.

Haplotype combination	CC	CT	TT
GG	GGCC	GGCT	GGTT
GA	GACC	GACT	GATT

### 3.17 Correlation analysis of haplotypes and cashmere performance in LCGs

According to Table 17, the haplotype combination with the best wool fineness performance in doe is GGTT, and it also performs the best in terms of wool length and wool yield, making it the overall optimal type. The overall optimal type in buck is the GGCC haplotype combination, which has advantages in shearing amount, fineness, length variation coefficient, and cashmere yield. The GACC haplotype combination has the best performance in terms of cashmere fineness.

**Table 17:** Correlation analysis of haplotypes and cashmere performance of LCGs.

Gender	Haplotype	Cashmere quantity	Fineness	Natural	Coefficient of variation	Number of	Short-fiber	Cashmere
		(g)	( µ m)	length (mm)	of length (%)	crimps	content (%)	rate (%)
Doe	GGCC(70/952)	1720.00 ± 30.36^cB^	16.37 ± 0.07^cB^	97.96 ± 2.14^aA^	51.99 ± 0.93^cC^	5.14 ± 0.52^cB^	0.14 ± 0.02^cdCD^	0.73 ± 0.01^cB^
	GGCT(56/952)	1812.50 ± 8.76^bB^	16.58 ± 0.13^bcB^	64.95 ± 2.37^bB^	60.86 ± 1.52^bB^	3.93 ± 0.57^dB^	0.33 ± 0.03^aA^	0.75 ± 0.01^bcB^
	GGTT(70/952)	1720.00 ± 10.50^cB^	15.72 ± 0.12^dC^	100.88 ± 4.39^aA^	68.01 ± 1.14^aA^	8.00 ± 0.60^aA^	0.24 ± 0.01^bB^	0.88 ± 0.01^aA^
	GACC(196/952)	1803.57 ± 20.72^bB^	16.38 ± 0.11^cB^	99.29 ± 2.15^aA^	49.69 ± 0.91^cdCD^	7.43 ± 0.23^abA^	0.11 ± 0.01^dD^	0.78 ± 0.01^bB^
	GACT(308/952)	1925.00 ± 18.19^aA^	17.11 ± 0.07^aA^	104.22 ± 1.92^aA^	46.89 ± 0.68^dD^	7.13 ± 0.22^abA^	0.12 ± 0.01^dD^	0.67 ± 0.01^dC^
	GATT(252/952)	1602.78 ± 18.33^dC^	16.79 ± 0.08^abAB^	96.08 ± 1.47^aA^	58.24 ± 0.62^bB^	6.76 ± 0.24^bA^	0.17 ± 0.01^cC^	0.75 ± 0.01^bcB^
Buck	GGCC(4/42)	2300.00 ± 0.00^aAB^	16.16 ± 0.87^bAB^	78.00 ± 3.58^b^	51.19 ± 7.08^b^	5.45 ± 3.15	0.29 ± 0.10^abAB^	0.79 ± 0.08^abAB^
	GGCT(4/42)	2425.00 ± 43.30^aA^	16.73 ± 0.77^abA^	94.90 ± 2.94^ab^	68.35 ± 1.55^a^	9.15 ± 0.38	0.13 ± 0.00^bB^	0.79 ± 0.08^abAB^
	GGTT(10/42)	1820.00 ± 37.42^bcCD^	16.81 ± 0.16^abA^	94.00 ± 6.69^ab^	52.69 ± 4.25^ab^	7.08 ± 1.19	0.14 ± 0.03^bB^	0.74 ± 0.02^bAB^
	GACC(6/42)	1583.33 ± 100.55^cD^	14.84 ± 0.08^cB^	79.87 ± 10.63^b^	59.04 ± 5.85^ab^	6.73 ± 2.15	0.43 ± 0.08^aA^	0.94 ± 0.01^aA^
	GACT(8/42)	1887.50 ± 142.91^bcBCD^	16.54 ± 0.33^abA^	104.80 ± 3.24^a^	57.82 ± 2.47^ab^	7.95 ± 0.28	0.15 ± 0.02^bB^	0.72 ± 0.04^bAB^
	GATT(10/42)	2150.00 ± 114.99^abABC^	17.47 ± 0.34^aA^	86.32 ± 3.53^ab^	59.75 ± 4.55^ab^	6.94 ± 1.16	0.16 ± 0.06^bB^	0.69 ± 0.06^bB^

Note: the expression method with no significant difference ( p>0.05 ) is the same shoulder letter, the expression method with a significant difference ( p<0.05 ) is the shoulder letter with different lowercase letters, and the expression method with an extremely significant difference ( p<0.01 ) is the shoulder letter with different uppercase letters.

### 3.18 Correlation analysis of haplotypes and milk performance of LCGs

According to Table 18, in LCG doe, the GGCT haplotype combination performs significantly better in Cru.Prot, SnF, TS, Cond., and H.Index compared to the other haplotypes. The GGTT haplotype shows excellent performance in fat, lactose, urea, N , TS, and H.Index. Based on correlation studies, it is known that fat, TS, and SnF are the main factors affecting cashmere fineness. Considering cashmere fineness comprehensively, the GGTT haplotype is identified as the optimal haplotype.

**Table 18:** Correlation analysis of haplotypes and milk performance of LCGs.

Haplotype	Fat	Cru.Prot	Lactose	Urea	N	SnF	TS	Cond.	H.Index
GGCC(42/273)	7.00 ± 0.31^bcAB^	4.41 ± 0.03^cC^	5.30 ± 0.03^aA^	40.82 ± 0.76^aA^	19.05 ± 0.35^aA^	10.17 ± 0.04^cC^	17.51 ± 0.34^cdBC^	755.90 ± 8.47^bB^	0.45 ± 0.01^bAB^
GGCT(42/273)	6.67 ± 0.43^bcB^	6.98 ± 0.47^aA^	4.56 ± 0.23^bB^	36.47 ± 1.36^bB^	17.02 ± 0.64^bB^	12.36 ± 0.37^aA^	19.51 ± 0.64^aA^	859.73 ± 37.32^aA^	0.50 ± 0.03^aA^
GGTT(77/273)	7.39 ± 0.29^abAB^	4.49 ± 0.05^cC^	5.28 ± 0.02^aA^	39.48 ± 0.44^aAB^	18.42 ± 0.21^aAB^	10.25 ± 0.05^cC^	18.00 ± 0.32^bcdABC^	758.22 ± 7.06^bB^	0.50 ± 0.01^aA^
GACC(21/273)	6.06 ± 0.32^cB^	4.65 ± 0.05^cC^	5.39 ± 0.03^aA^	40.03 ± 1.12^aA^	18.67 ± 0.52^aA^	10.58 ± 0.05^cBC^	16.95 ± 0.35^dC^	739.87 ± 8.54^bB^	0.39 ± 0.01^bB^
GACT(49/273)	8.32 ± 0.24^aA^	4.57 ± 0.06^cC^	5.19 ± 0.03^aA^	41.16 ± 0.58^aA^	19.20 ± 0.27^aA^	10.27 ± 0.05^cC^	18.99 ± 0.26^abAB^	742.14 ± 6.98^bB^	0.50 ± 0.01^aA^
GATT(42/273)	7.10 ± 0.33^bcAB^	5.58 ± 0.35^bB^	5.07 ± 0.11^aA^	41.62 ± 0.90^aA^	19.42 ± 0.42^aA^	11.28 ± 0.28^bB^	18.73 ± 0.46^abcAB^	767.47 ± 10.34^bB^	0.44 ± 0.01^bAB^

Note: the expression method with no significant difference ( p>0.05 ) is the same shoulder letter, the expression method with a significant difference ( p<0.05 ) is the shoulder letter with different lowercase letters, and the expression method with an extremely significant difference ( p<0.01 ) is the shoulder letter with different uppercase letters.

## 4 Discussion

LCGs are the highest-producing white cashmere goat breed in the world. They have long cashmere fibers, a high net cashmere rate, moderate cashmere fineness, pure white fur, a large body size, strong adaptability, stable genetic performance, and a good improvement effect on medium- and low-yield cashmere goats. They are known as the national treasure of China. With LCGs as the original breed, five new local breeds have been cultivated, making outstanding contributions to the improvement and cultivation of Chinese cashmere goat breeds. This article mainly conducted research on reducing the fineness of cashmere. There are many genes that affect the fineness traits of cashmere, such as *COL6A5* (Zhang et al., 2021), *FA2H* (Wu et al., 2022a), *COL1A1* (Ma et al., 2023), and *BAAT* (Cai et al., 2023). This study primarily explored the impact of SNP sites within *KRT27* and *ELOVL4*, along with their haplotype combinations, on cashmere yield and lactation traits. We detected the 1919G/A site of *KRT27* and the 28666C/T site of *ELOVL4* separately. We utilize PIC, $H_{e}$ , and $N_{e}$ as metrics to assess the level of genetic diversity within the population. PIC values exceeding 0.5 signify a high level of polymorphism, those ranging from 0.25 to 0.5 indicate moderate polymorphism, and those below 0.25 suggest a low level of polymorphism. The lower the $H_{e}$ value, the lower the genetic variability. The $N_{e}$ value indicates the maintenance ability of its allele in the population and mutants. The PIC values of the 1919G/A locus of *KRT27* and the 28666C/T locus of *ELOVL4* in both male and female LCGs were between 0.25 and 0.5, indicating moderate polymorphism in the population, with a $\chi^{2}$ value > 1 and a p value < 0.05, which does not meet the hypothesis of the Hardy–Weinberg equilibrium. This result may be due to the intensive artificial selection of goats on the breeding farm, leading to widespread gene flow.

The *KRT* family plays a significant role in the formation of keratinocytes in animals. *KRT1* was discovered by Jin et al. (2016). *KRT26* is involved in the regulation of cashmere development. *KRT6/16/17* is a key early barrier warning protein (Zhang et al., 2019). *KRT79* is involved in the formation of hair ducts (Veniaminova et al., 2013). The *ELOVL* family plays a crucial role in maintaining cellular lipid metabolism balance, skin barrier function, and metabolic and inflammatory regulation. *ELOVL1* is related to the pathogenesis of ichthyosis and other diseases (Mueller et al., 2019). *ELOVL5* elongates n-3 and n-6 polyunsaturated fatty acids, affecting the lipid composition in serum (Tomida et al., 2021). The expression level and activity of *ELOVL6* are linked to metabolic disorders, including obesity and type-2 diabetes (Macášek et al., 2021). Currently, there is limited research on *KRT27* and *ELOVL4*. We found through SNP experiments that mutations at the 1919G/A locus of *KRT27* and the 28666C/T locus of *ELOVL4* both affect cashmere fineness, and they also have varying degrees of impacts on other production performances of LCGs. Of these, the GG genotype of 1919G/A shows excellent performance and dominates the cashmere fineness, body size, slaughter, and meat quality of LCGs in both male and female goats, while the 28666C/T locus is more complex. The CC type has advantages in cashmere fineness, body size performance, lactation performance, slaughter performance, protein content, and fat content in LCGs and does, while it has advantages in cashmere fineness, body size performance, and slaughter performance in buck. The CT type is superior in terms of wool-cutting volume, tube circumference, lambing performance, and lactation performance in doe, while ram wool-cutting volume, body length, carcass weight, eye muscle area, and protein content are superior. The TT type is relatively balanced, with outstanding advantages in cashmere fineness and lactation performance in doe and outstanding advantages in body size, eye muscle area, and fat content in buck. Based on the overall fineness and lactation performance of cashmere goats, the 28666C/T locus TT type of *ELOVL4* is dominant. Through haplotype combination of two genes, we found that the haplotype combination GGTT is the dominant haplotype combination with multiple factors and effects in cashmere fineness, and it also has an advantage in lactation performance.

The fineness of cashmere, as an important indicator of cashmere quality, is related to the texture, comfort, and warmth of the textile. Due to its regulation by multiple factors, the variation of cashmere fineness is difficult to predict. A study has found that the body size of LCGs has a certain impact on the fineness of cashmere (Meng et al., 2022). Yifei et al. (2024) analyzed the correlation between cashmere fineness and the wool and cashmere of Yanshan cashmere goats and established the optimal regression equation. Therefore, correlation analysis of other production data and cashmere fineness may reveal the factors affecting cashmere fineness. This study aimed to establish the relationship between wool production performance and wool fineness, establish the relationship between lactation performance and wool fineness in LCGs, investigate the correlation between wool production performance and wool fineness, and investigate the correlation between lactation performance and wool fineness. The results showed that there was a highly significant correlation between cashmere yield, shearing yield, length variation coefficient, length, and cashmere fineness in the cashmere production performance of LCGs. There was also a highly significant correlation between TS, urea, and cashmere fineness in the lactation performance, and an optimal linear regression model was established using the highly significant traits.

As a valuable germ-plasm resource in China, the issue of reducing cashmere fineness in LCG goats needs attention. This study genetically analyzed the production performance of SNP loci within *KRT27* and *ELOVL4*. Through comprehensive evaluation, the optimal haplotype combination GGTT and the beneficial one-gene multi-effect genotype TT were selected. These findings contribute to the breeding and enhancement of LCGs, advance the genetic breeding of cashmere goats, and offer theoretical support for cultivating superior cashmere goat varieties.

## 5 Conclusion

In this study, *KRT27* had a SNP locus, 1919G/A, where the GG genotype excelled in wool fineness among LCG doe, while the GA genotype was superior in LCG buck. Additionally, a SNP locus, 28666C/T, was identified in *ELOVL4*, with the CC genotype demonstrating outstanding cashmere fineness. The advantageous haplotype combination with multiple factors and effects of cashmere fineness was determined to be the GGTT haplotype combination, and then correlation analysis was conducted of the phenotype of cashmere fineness with cashmere production performance and lactation performance. Through multiple linear regression (MLR) analysis, it was found that the trait with the greatest direct impact on cashmere production performance and fineness is the cashmere yield rate, with a correlation coefficient of 0.915. The trait with the greatest direct impact on milk production performance and cashmere fineness is TS, with a correlation coefficient of 16.369. The dominant genotype that affects the fineness and associated traits of cashmere goats has been identified as the TT genotype.

## Data Availability

The data sets used in this article can be requested from the corresponding author.
